# Protection against invasive meningococcal disease and vaccination policy in the Netherlands

**DOI:** 10.1093/eurpub/ckac130.098

**Published:** 2022-10-25

**Authors:** M Ohm, M Knol, E Sanders, G Berbers

**Affiliations:** Centre for Infectious Disease Control, RIVM, Bilthoven, Netherlands

## Abstract

**Background:**

A rise in serogroup C invasive meningococcal disease (IMD-C) led to introduction of MenC vaccination in 2002 in the Netherlands at 14 months of age, accompanied by a mass-campaign for all children between 1 and 18 years (coverage 94%). Due to an IMD-W outbreak in 2016-17, the MenC vaccine was replaced by a MenACWY vaccine and an adolescent booster at 14 years was introduced next to a mass campaign for 14-18 year-olds in 2018.

**Aim/methods:**

We explored meningococcal antibody status in the Netherlands across the population in 2006-07, 2016-17 and 2020 in consecutive cross-sectional serosurveillance studies. Furthermore, we assessed the vaccine impact and effectiveness of the recent MenACWY vaccination campaign. We determined long-term protection in both adolescents and adults after a MenACWY vaccination and investigated sex-related differences in the vaccine response in adolescents.

**Results:**

MenC antibody levels were low in 2016-17, except in recently vaccinated toddlers and individuals who were vaccinated as teenagers in 2002. We demonstrated waning of MenC immunity 15 years after the mass campaign and highlighted the lack of meningococcal AWY immunity across the population, which underlined the importance of the recently introduced MenACWY (booster) vaccination. The MenACWY vaccination program was effective in preventing IMD-W in the target population. Long-term protection was achieved for MenC, MenW, and MenY in 94-96% of adolescents five years postvaccination, but in adults only in 32%, 65% and 71% for MenC, W and Y. Adolescent antibody responses were higher in girls than in boys for all serogroups at most timepoints after MenACWY vaccination. The differences in average titers were however small and the percentage of participants with protective titers was very high for both sexes.

**Conclusions:**

The current meningococcal vaccination policy in the Netherlands provides protection across the population against IMD-ACWY and seems sufficient on the long-term.

**Key messages:**

- The current meningococcal vaccination policy in the Netherlands provides protection across the population against IMD-ACWY and seems sufficient on the long-term.
- The current meningococcal vaccination policy in the Netherlands provides protection across the population against IMD-ACWY and seems sufficient on the long-term.

